# Supplementary material for: GIGANTEA Is Required for Robust Circadian Rhythms in Wheat
Source: Plant Cell Environ. 2025 Feb 26;48(6):4492–504. doi: 10.1111/pce.15447 (PMC12050397; doi:10.1111/pce.15447)
Supplement: Supplementary file 2 — Supporting information. [file PCE-48-4492-s003.docx]

Supplementary Table S1

| **Variety Name** | **Locality** | **Species** | **Habit** | **Assembly** |
| --- | --- | --- | --- | --- |
| Arina | Switzerland | *T. aestivum* | W | De novo |
| CDC Stanley | Canada | *T. aestivum* | S | De novo |
| CDC Landmark | Canada | *T. aestivum* | S | De novo |
| Claire | UK | *T. aestivum* | W | W2RAP |
| Cadenza | UK | *T. aestivum* | F | W2RAP |
| Jagger | USA | *T. aestivum* | W | De novo |
| Julius | Germany | *T. aestivum* | W | De novo |
| Norin61 | Japan | *T. aestivum* | S | De novo |
| Mace | Australia | *T. aestivum* | S | De novo |
| Robigus | UK | *T. aestivum* | W | W2RAP |
| PI190962 | Europe | *T. spelta* | W | / |
| Paragon | UK | *T.aestivum* | S | W2RAP |
| Lancer | Australia | *T.aestivum* | S | W2RAP |
| SY Mattis | France | *T. aestivum* | W | De novo |
| Zativan | Israel | *T. turgidum* | NA | De novo |

Supplementary Table S2

|  | **Genotype** | **NPQ ± SEM** | **Fv/Fm ± SEM** |
| --- | --- | --- | --- |
| **Period** | Kronos | 23.30 ± 0.28 | 22.70 ± 0.20 |
|  | WT Segregant | 22.70 ± 0.27 | 23.10 ± 0.14 |
|  | *Ttgi-B3* | 22.70 ± 0.22 | 22.05 ± 0.11 |
|  | *Ttgi-A3* | 21.30 ± 0.14 | 21.70 ± 0.11 |
|  | *Ttgi-A3/gi-B3* | 18.40 ± 0.38 | 20.80 ± 1.14 |
| **RAE** | Kronos | 0.20 ± 0.01 | 0.28 ± 0.01 |
|  | WT Segregant | 0.17 ± 0.01 | 0.18 ± 0.02 |
|  | *Ttgi-B3* | 0.21 ± 0.01 | 0.26 ± 0.02 |
|  | *Ttgi-A3* | 0.26 ± 0.01 | 0.27 ± 0.02 |
|  | *Ttgi-A3/gi-B3* | 0.59 ± 0.05 | 0.52 ± 0.04 |
