# Supplementary material for: GIGANTEA Is Required for Robust Circadian Rhythms in Wheat
Source: Plant Cell Environ. 2025 Feb 26;48(6):4492–504. doi: 10.1111/pce.15447 (PMC12050397; doi:10.1111/pce.15447)
Supplement: Supplementary file 4 — Supporting information. [file PCE-48-4492-s001.docx]

**Supplementary data**

**Supplementary Figure S1.** The expression of *TtGI* is consistent across both subgenomes with no significant differences between the wild type genotypes and the double mutant. *Ttgi-A3, Ttgi-B3,* *Ttgi-A3/gi-B3*, Kronos and WT segregant (n▒=▒4-5) grown under long day (16▒h light at 250 µmol m^−2^ s^−1^, 20°C: 8▒h dark 16°C). Expression of *TtGI* was measured relative to *TtRPT5A* and *Ta22845* at ZT12 and ZT15 (mean**▒**±▒SEM)***.*** The letters within the panel indicate statistical difference, samples that share the same letter in that experiment are not significantly different.

**Supplementary Figure S2.** Isolation of TILLING mutants in tetraploid wheat. Crossing scheme for the creation of *Ttgi* single mutants (A), single mutant *Ttgi-A3* and (B) single mutant *Ttgi-B3.* Wild type gene is designated by a capital letter and mutated gene with lower letter. The TILLING lines *GI* Kronos2019 and Kronos2205 were crossed with Kronos background (F1). Four rounds of back crossing were completed and plants heterozygous (Aa/Bb) for the mutation selected. BC4 F1 was self-crossed to obtain BC4 F2, which was self-crossed again. Homozygous plants (aa and bb) for the mutation selected for single subgenome genotype (*Ttgi-A3* and *Ttgi-B3*, respectively) were selected. The single mutants were crossed (BC4 F4) and the progeny was self-crossed. From which the double mutants and background segregants *Ttgi-A3/gi-B3* [aabb], WT segregant [AABB]) were selected.

**Supplementary Figure S3.** Overview of the field trial used to evaluate *TtGI* lines. A) Photograph of trial at NIAB Barr Hill field site, Cambridge, U.K). Plots of Kronos *GI* lines (awned) and Paragon controls (no awns) were flanked by Paragon plots to separate the *GI* experiment from other trials. B) Layout of trial for 2021 field season. Six plots of each genotype were drilled in a randomized block design (Paragon was not used for this analysis). Each genotype featured once in each row and a maximum of twice in each column.

**Supplementary Figure S4.** The chlorophyll *a* parameter, *Fv/Fm* is also arrhythmic in *Ttgi-A3/gi-B3* in constant conditions (light and temperature) supporting data from NPQ parameter (Figure [2](#MEP_L_fig2)). Mean of *Fv/Fm* (± SEM) of Kronos (A), WT Segregant (B), *Ttgi-B3* (C), *Ttgi-A3* (D) and *Ttgi-A3/gi-B3* (E) (n▒=▒> 10). White bars represent the subjective day and grey bars represent the subjective night. (F) Circadian period length (hours). (G) Relative Error of Amplitude (RAE). A RAE value above 0.5 (indicated by the dashed line) is considered arrhythmic. Period and RAE of *Fv/Fm* were calculated using Biodare2. Significant differences (P▒<▒0.05) calculated in R using the Kruskal–Walli’s test followed by post-hoc Dunn’s test. (F) and (G) **t**he letters within each panel indicate statistical difference, samples that share the same letter in that experiment are not significantly different.

**Supplementary Figure S5.** *TtGI* lines and Kronos produced equivalent yields in controlled long photoperiod. Kronos, WT segregant, *Ttgi-A3*, *Ttgi-B3* and *Ttgi-A3/gi-B3* plants were grown under long photoperiod (LD, 16▒h light at 250 µmol m^−2^ s^−1^, 20°C: 8▒h dark 16°C). (A) Plant length. (B) Number of tillers. (C) Length of the primary head. (D) Number of seeds per primary head. (E) Weight of seeds produced by the primary head. (F) Total number of seeds produced by each plant. Each jitter point represents an individual plant (n▒=▒12). Significant differences were tested for using either an ANOVA with a post hoc Tukey test (plant height, primary head length, weight of seeds) or Kruskal Wallis with a post hoc Dunn test (tiller number, seed number). The letters within each panel indicate statistical difference, samples that share the same letter in that experiment are not significantly different.

**Supplementary Figure S6.** Morphological traits were broadly similar between *GI* lines and Kronos plants. Absence of a functional *TtGI* had a significantly reduced yield in controlled short photoperiod compared to Kronos. Kronos, WT segregant, *Ttgi-A3*, *Ttgi-B3* and *Ttgi-A3/gi-B3* plants were grown under short photoperiod (SD, 8▒h light at 250 µmol m^−2^ s▒−▒^1^, 20°16: 16▒h dark 16°C). (A) Plant length. (B) Number of tillers. (C) Length of the primary head. (D) Number of seeds per primary head. (E) Weight of seeds produced by the primary head. (F) Total number of seeds produced by each plant. Each jitter point represents an individual plant (n▒=▒8). Significant differences were tested for using either an ANOVA with a post hoc Tukey test (plant height, primary head length, weight of seeds) or Kruskal Walli’s with a post hoc Dunn’s test (tiller number, seed number). The letters within each panel indicate statistical difference, samples that share the same letter in that experiment are not significantly different.

**Supplementary Figure S7.** Morphological and yield traits were similar between the *TtGI* lines and Kronos in field conditions. Kronos, WT segregant, *Ttgi-A3*, *Ttgi-B3* and *Ttgi-A3/gi-B3* plants were grown in NIAB experimental farm during the 2020 field season. (A) Height. (B) Length of primary head. (C) Number of seeds of representative head. (D) Mean weight of seeds of representative head per plot. Significant differences were tested for using either an ANOVA with a post hoc Tukey test (plant height, primary head length, weight of seeds) or Kruskal Wallis with a post hoc Dunn test (seed number). The letters within each panel indicate statistical difference, samples that share the same letter in that experiment are not significantly different.

**Supplementary Figure S8. Mutation** of function of *TtGI* did not affect the abundance of transcripts expression of genes involved in gibberellin synthesis. *Ttgi-A3/gi-B3* (yellow, n▒=▒4-5) and Kronos WT (black, n▒=▒4-5) grown under long day (16▒h light at 250 µmol m▒−▒2▒s▒−▒1, 20°C: 8▒h dark 16°C). Once plants reached 3-leaves or GS39, sampling of the first true leaf commenced at time 0 to 24▒hours every 3▒hours. White bars represent light and black bars represent darkness. (A-F) Mean abundance (▒±▒SEM, represented by the shaded ribbon) of flowering at the 3-leaf growth stage. (G-L) Mean abundance (▒±▒SEM, represented by the shaded ribbon) of flowering at the GS39 growth stage. Transcript abundance (ΔΔCq) is relative to *TtRP15* and *TtRPT5A*, (A-C) *TtGID1*, (B-D) *TtGA20ox2*

**Supplementary Table S1.** Wheat genome sequences sequenced by the wheat community (Avni et al., 2017; Walkowiak et al., 2020). Zativan (wild emmer, *T. turgidum sub spp. dicoccoides*) sequence available from Avni et al. 2017. All other sequences available from [www.10wheatgenomes.com](http://www.10wheatgenomes.com) sequence portal and Ensembl Plants. Information for this table was gathered from [www.10wheatgenomes.com](http://www.10wheatgenomes.com) and Adamski et al., 2020. S= spring, F= facultative and W= winter growth habit.

**Supplementary Table S2**. *Ttgi-A3/gi-B3* is arrhythmic for chlorophyll *a* fluorescence. *Ttgi-A3/gi-B3* is arrhythmic for chlorophyll *a* fluorescence. Period and RAE (Average ± SEM) of NPQ and *Fv/Fm* calculated using Biodare2 from Kronos, WT segregant, *Ttgi-B3*, *Ttgi-A3* and *Ttgi-A3/gi-B3* leaf fragments in constant light (n▒=▒> 10). A RAE value above 0.5 is considered arrhythmic.
