# Supplementary material for: GIGANTEA Is Required for Robust Circadian Rhythms in Wheat
Source: Plant Cell Environ. 2025 Feb 26;48(6):4492–504. doi: 10.1111/pce.15447 (PMC12050397; doi:10.1111/pce.15447)

Supplementary Figure 1

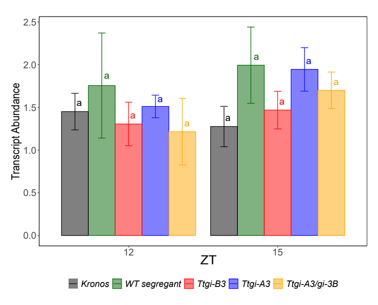

Supplementary Figure 2

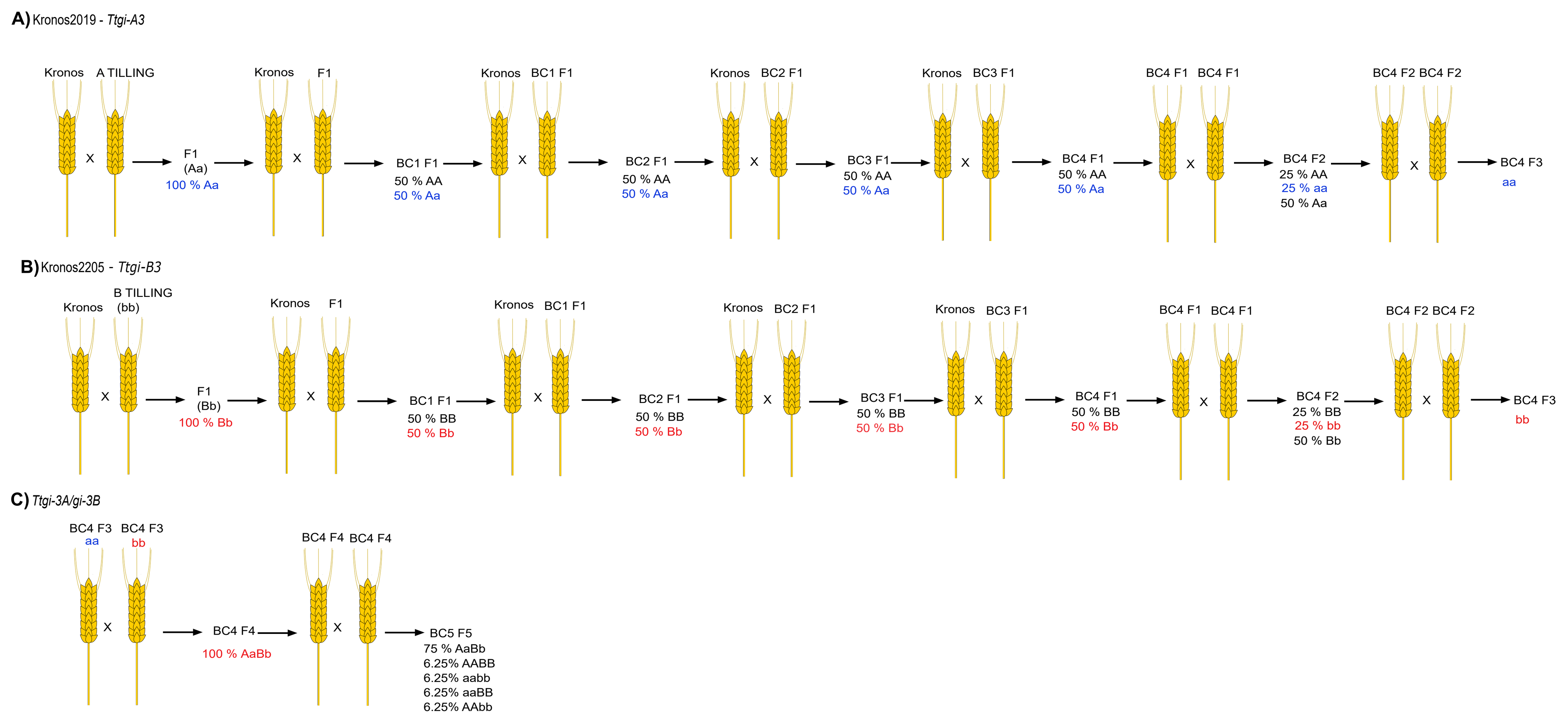

Supplementary Figure 3

A)

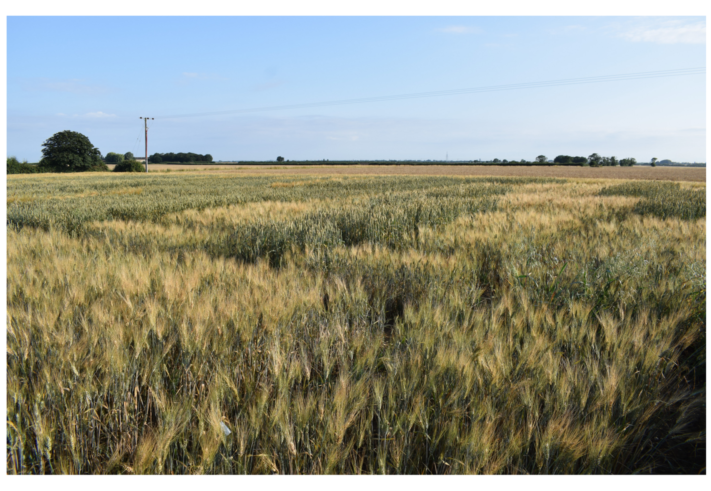

B)

Block/Column	1	2	3	4	5
1	Kronos	*Ttgi-A3/gi-B3*	WT segregant	*Ttgi-A3*	Paragon
2	*Ttgi-A3/gi-B3*	*Ttgi-A3*	Paragon	Kronos	WT segregant
3	Paragon	*Ttgi-A3*	Kronos	WT segregant	*Ttgi-A3/gi-B3*
4	*Ttgi-A3*	WT segregant	Kronos	Paragon	*Ttgi-A3/gi-B3*
5	*Ttgi-A3/gi-B3*	Kronos	Paragon	WT segregant	*Ttgi-A3*
6	Paragon	WT segregant	*Ttgi-A3/gi-B3*	*Ttgi-A3*	Kronos

Supplementary Figure 4

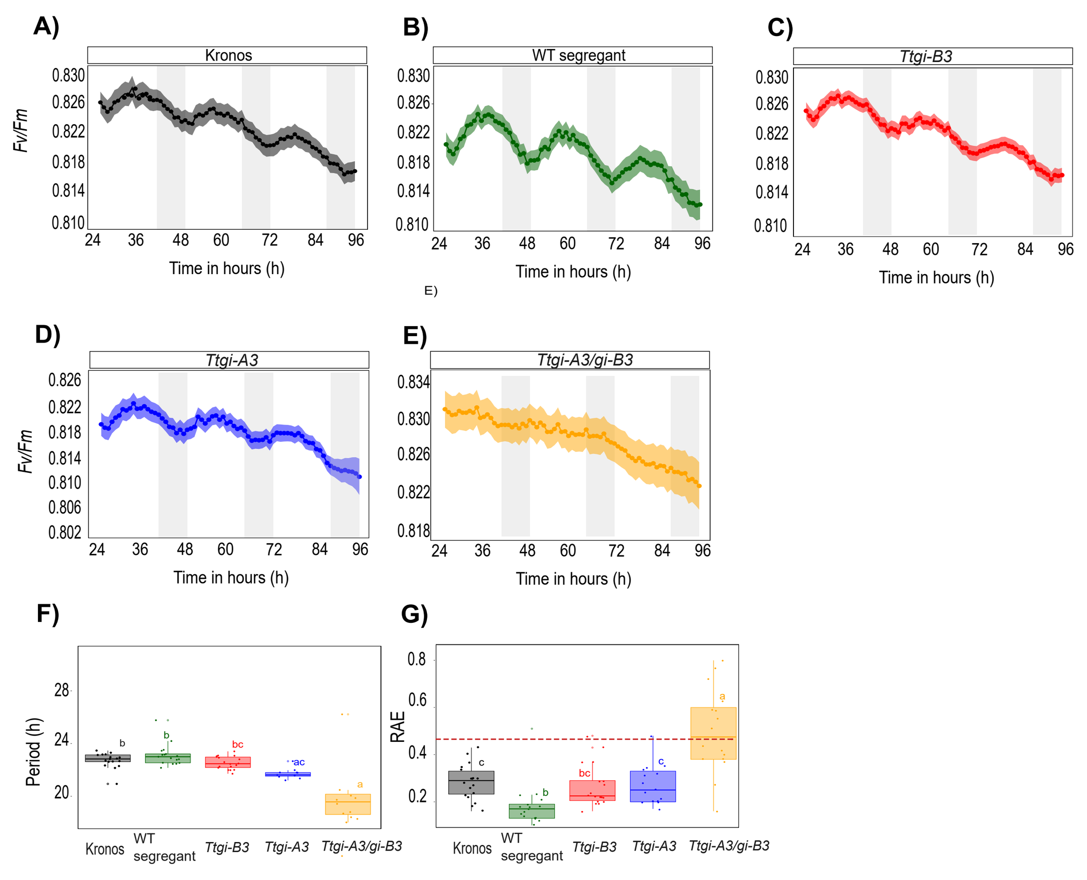

Supplementary Figure 5

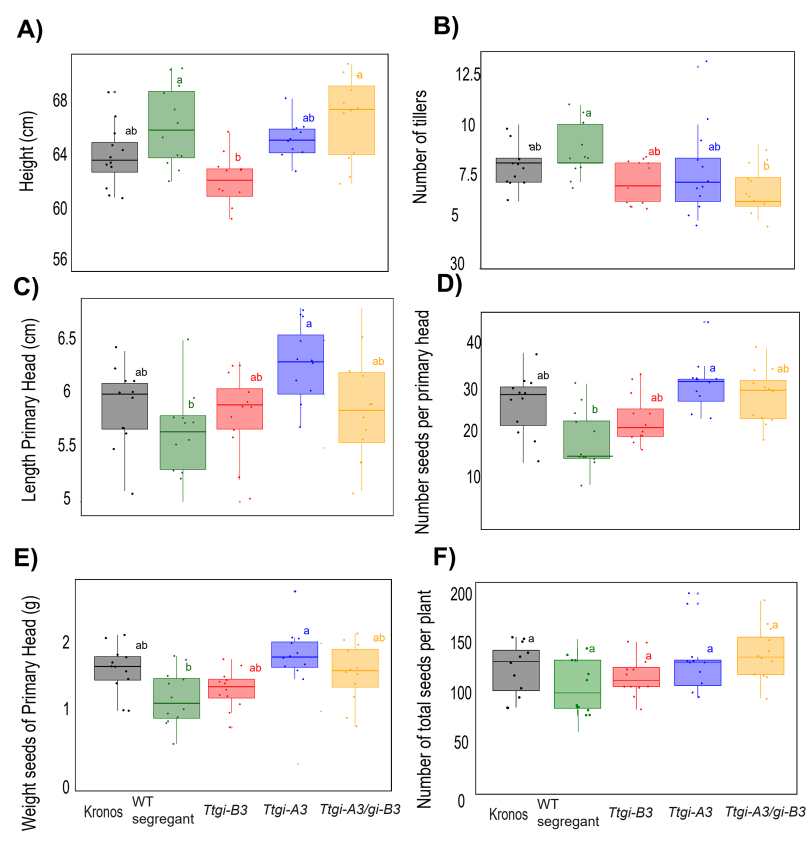

Supplementary Figure 6

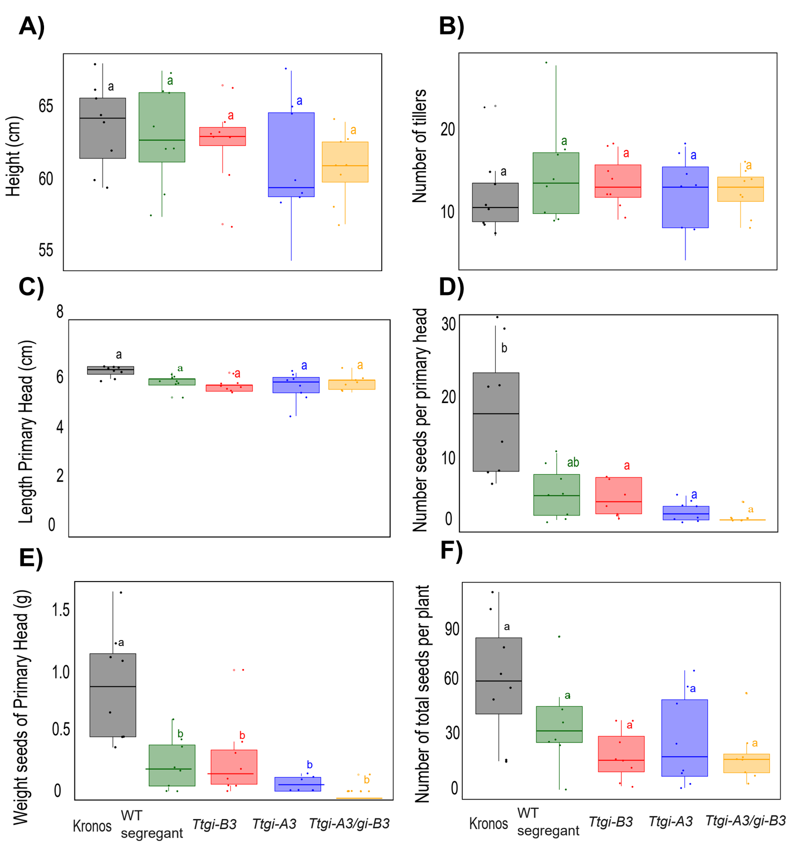

Supplementary Figure 7

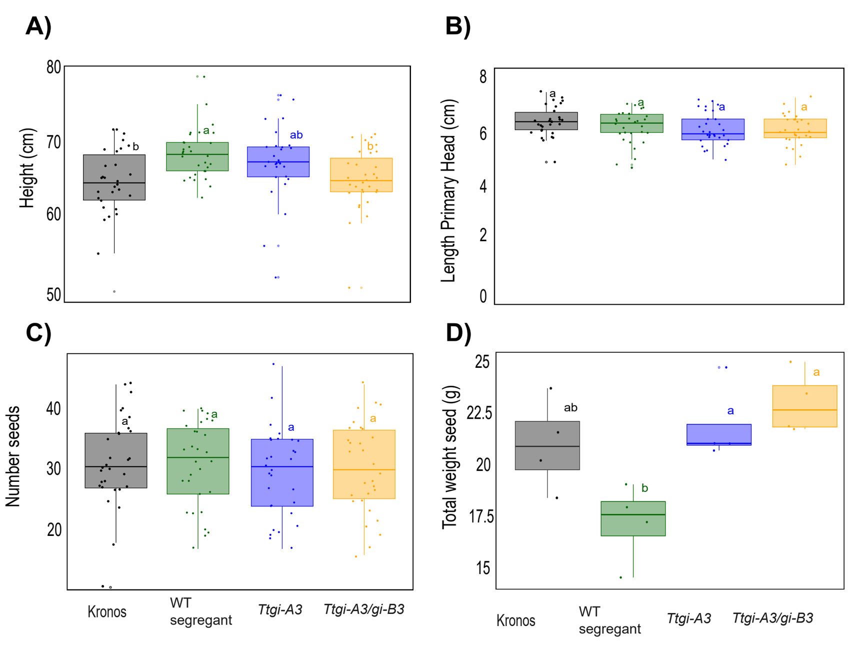

Supplementary Figure 8

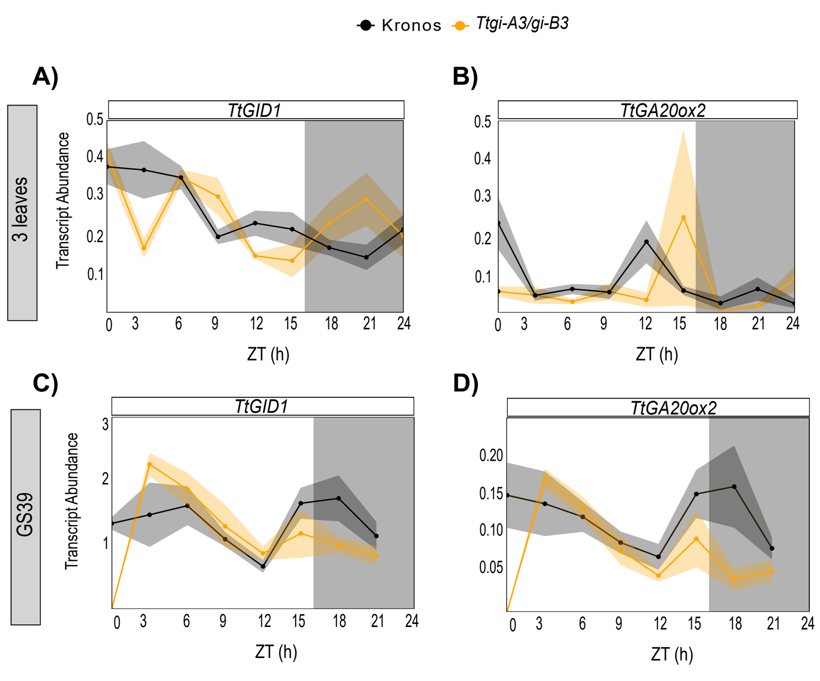

Supplement: Supplementary file 3 — Supporting information. [file PCE-48-4492-s004.pdf]
